# Phenotypic, Physiological, and Transcriptomic Analyses Reveal Different Responses to Salt Stress in Cultivated Red Lettuce and Wild Lettuce Seedlings

**DOI:** 10.3390/ijms26073425

**Published:** 2025-04-06

**Authors:** Wei Chen, Jiahao Lian, Caiyun Hong, Shuguang Sun, Jia Hao, Shengqi Huang, Jialin Wang, Yue Guan, Zhenwei Lu, Zhenlong Wang, Shixin Zhu, Zhen Wei

**Affiliations:** 1 School of Life Sciences, Zhengzhou University, Zhengzhou 450001, China; 2 National Innovation Centre for Bio-Breeding Industry, Xinxiang 453519, China

**Keywords:** domesticated lettuce, *Lactuca serriola*, salinity, phenylpropanoids, isoflavonoids

## Abstract

Cultivated lettuce (*Lactuca sativa* L.) is considered one of the most important economic vegetables worldwide; however, it is subjected to different stresses (salt stress, etc.) during its growth and development, resulting in yield reductions. In this study, we selected cultivated red lettuce and wild lettuce species (*Lactuca serriola* L.) to investigate the phenotypic and physiological changes in these lettuce under different salt treatment conditions. Functional annotation and enrichment analysis of the differentially expressed genes (DEGs) in the lettuce leaves and roots between the control and salt treatments were performed, identifying the key genes responding to salt stress. The results showed that the growth of the two types of lettuce was limited by salt stress, with decreased leaf area, main root length, biomass, and photosynthesis parameters noted. The cultivated red lettuce and the wild lettuce exhibited similar trends in terms of the variation in their antioxidant enzymatic activity and the content of osmoregulatory compounds in their leaves. The results of our transcriptomic analysis revealed that the mitogen-activated protein kinase (MAPK) signaling pathway, transporters, cytochrome P450, phenylpropanoid biosynthesis, and isoflavonoid biosynthesis were involved in the response to salt stress in the lettuce seedlings. The red lettuce cultivar showed a greater abundance of DEGs related to secondary metabolite biosynthesis and aquaporins under the salt treatment, resulting in a salinity-tolerant capacity comparable to that of the wild lettuce species. These results reveal important biosynthesis pathways that may play a key role in the salt tolerance of lettuce seedlings and provide key candidate genes that could be functionally characterized further and utilized to genetically improve new salt-tolerant varieties.

## 1. Introduction

Salt stress is one of the most common abiotic stresses in agricultural production worldwide [1]. In recent years, the worldwide distribution of saline lands and the salinization of non-saline land have significantly reduced the total area of arable land, posing a serious threat to the ecological environment and agricultural production [2]. The damage caused by salt stress can be divided into two stages: osmotic stress and ion toxicity. In the early stages of salt stress, the accumulation of salt in the soil affects the osmotic balance in plants and reduces their water absorption capacity. Subsequently, ion toxicity is induced by the salt ions accumulated in the plant cells, hindering intracellular metabolic processes [3]. The physiological and biochemical characteristics and gene expression (*HKT*, *NHX*, *SOS*, etc.) of the plants are adjusted to adapt to changes in their living environment to enable them to maintain intracellular sodium and potassium homeostasis through Na^+^ efflux and K^+^ influx control [4,5,6]. Plants can also enhance the activity of antioxidant enzymes, such as superoxide dismutase, peroxidase, and glutathione transferase, to scavenge the excess intracellular reactive oxygen species (ROS) generated during salt stress [7]. In addition, plants can alleviate osmotic stress by enhancing the synthesis of osmoregulatory compounds, such as proline and sugar alcohol [8]. Phytohormones, for example, abscisic acid (ABA), jasmonic acid (JA), and salicylic acid (SA), also play a pivotal role in the response of plants to abiotic stresses, including salt stress [9,10,11]. Understanding the regulatory mechanisms of the salt stress response and identifying salt-resistance genes in plants represent vital steps in improving salt tolerance in crop plants and increasing their production.

Cultivated lettuce (*Lactuca sativa* L.) is an important economic vegetable around the world, and taxonomically, it belongs to *Lactuca* L. (Asteraceae) [12]. As vegetables, lettuce cultivars are considered healthy dietary sources of minerals, fibers, vitamins, and antioxidant compounds [13,14]. Based on statistics from the Food and Agriculture Organization of the United Nations, the worldwide gross production value of lettuce and chicory in 2023 was USD 16.26 billion. Among the different leafy vegetables worldwide, lettuce and chicory rank second and third, respectively, in terms of the size of the area harvested and their production, with China, India, the U.S.A., Spain, and Italy ranking in the top five in terms of the area harvested and the production of lettuce and chicory. The gross yield of lettuce and chicory in China is greater than that of all other countries combined (FAOSTAT https://www.fao.org/faostat/en/#home, accessed on 24 March 2025). It is evident from the above that cultivated lettuce is a very popular, widely consumed leafy vegetable of great economic value around the world.

Cultivated lettuce is salt-sensitive, and the germination and growth of its seeds are impaired under salt stress [15]. Authors of recent studies on salt stress in lettuce have focused on how exogenous chemicals, bio-stimulants, and phytohormones can enhance its antioxidant capacity or photosynthetic properties [16,17]. *Lactuca serriola* L. is a wild relative of *L. sativa* and is considered a genetic resource for lettuce breeding [18]. In one study, an RIL population developed from *L. sativa* and *L. serriola* was used to identify quantitative trait loci (QTLs) related to salt stress [19]. Despite the above advances in the research, the gene expression, antioxidant capacity, and photosynthetic rates of lettuce in response to salinity stress have yet to be reported. In this study, we aim to investigate the different responses of cultivated and wild lettuce plants to salt stress using phenotypic, physiological, and RNA-seq analyses. The results highlight the key candidate genes responsive to salt stress in lettuce that could be functionally characterized further and utilized for the genetic improvement of new salt-tolerant varieties for lettuce breeding.

## 2. Results

### 2.1. Variations in the Phenotypic Traits of Lettuce During Salt Stress Treatment

**Figure 1:**
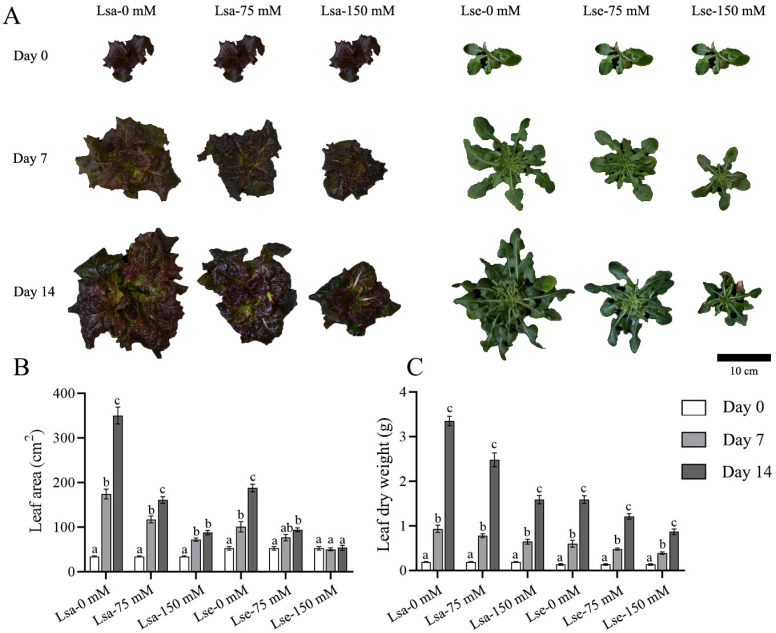
Phenotypic variations in the leaves of cultivated and wild lettuce in response to salt treatments for 14 days. Lsa is *L. sativa* ‘Lollo Rosso’; Lse is *L. serriola*. (**A**) Leaf trait variations in lettuce under 0, 75, and 150 mM NaCl treatments. The three columns on the left show cultivated lettuce Lsa, and the three columns on the right show wild lettuce Lse. (**B**) Leaf area variations in Lsa and Lse in response to 0, 75, and 150 mM NaCl treatments. (**C**) Dry weight of Lsa and Lse leaves in response to 0, 75, and 150 mM NaCl treatments. Different letters represent a significant difference between samples (*p* < 0.05). Error bars represent SE.

**Figure 2:**
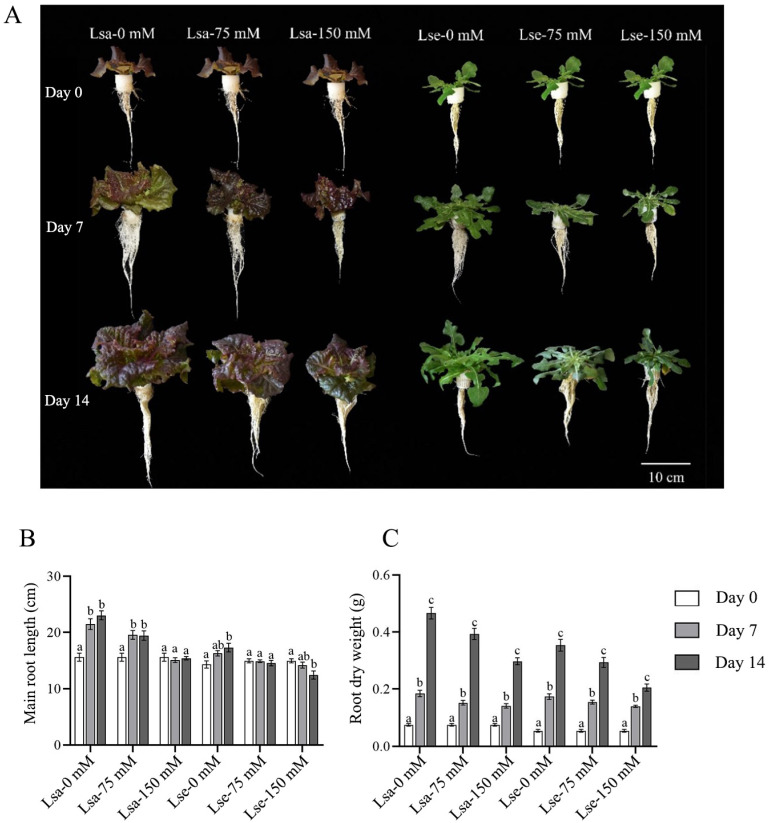
Phenotypic variations in the roots of cultivated and wild lettuce in response to salt treatment for 14 days. Lsa is *L. sativa* ‘Lollo Rosso’; Lse is *L. serriola*. (**A**) Root trait variations in lettuce under 0, 75, and 150 mM NaCl treatments. The three columns on the left show cultivated lettuce Lsa, and the three columns on the right show wild lettuce Lse. (**B**) Main root length variations in Lsa and Lse in response to 0, 75, and 150 mM NaCl treatments. (**C**) The dry weight of the Lsa and Lse roots in response to 0, 75, and 150 mM NaCl treatments. Different letters represent a significant difference between samples (*p* < 0.05). Error bars represent SE.

The phenotypic traits of lettuce seedlings in response to salt stress, including leaf area (LA), leaf dry weight (LDW), main root length (MRL), and root dry weight (RDW), were assessed on Day 0, Day 7, and Day 14 during different salinity treatments. The growth of the Lsa and Lse leaves and the roots were inhibited under salt stress, and the inhibition became more pronounced with an increasing NaCl concentration (Figure 1 and Figure 2).

After one week of treatment, the LA, LDW, and RDW of Lsa increased significantly compared with these values on Day 0 under the control and NaCl treatments. The MRL of Lsa increased significantly compared with that on Day 0 under the 0 mM and 75 mM NaCl treatments; however, growth was not observed when using 150 mM NaCl (Figure 1 and Figure 2). Moreover, the LA of Lse showed significant growth from Day 0 to Day 7 under the 0 mM treatment only, with a slow increase under the 75 mM treatment and no observed change under the 150 mM treatment. The MRL of Lse only increased under the 0 mM NaCl treatment. The LDW and RDW of Lse increased significantly compared with these values on Day 0 under all three treatments.

After two weeks of salinity stress (Day 14 compared with Day 7), the LA of Lsa increased significantly under the 0 mM and 75 mM NaCl treatment only and did not change significantly under the 150 mM NaCl treatment. The MRL did not change significantly under the three treatments; in comparison, the LDW and RDW increased significantly under all three treatments. The MRL of Lse did not increase under any of the treatments; the other growth parameters of Lse showed similar trends to those observed in the first week of treatment.

### 2.2. Physiological Changes in the Photosynthetic Traits, Antioxidant Enzymatic Activity, and Proline and Soluble Sugar Contents of Wild and Cultivated Lettuces Under the Salt Treatments

**Figure 3:**
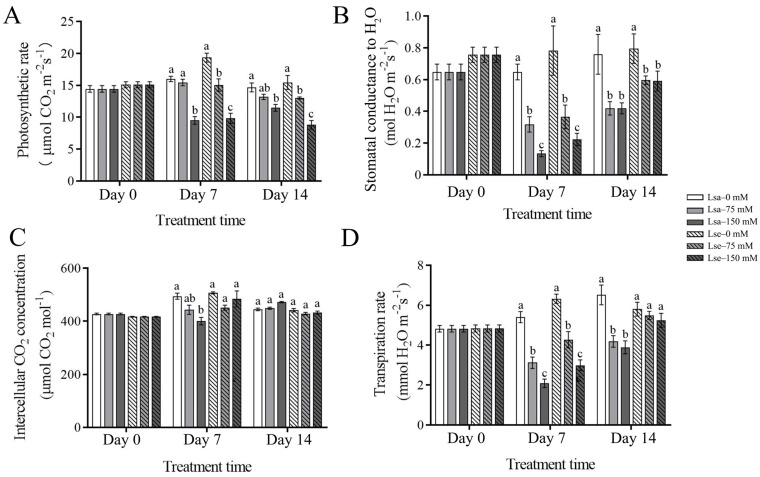
Variations in photosynthesis-related indexes under different treatments. Lsa is *L. sativa* ‘Lollo Rosso’; Lse is *L. serriola*. (**A**) Photosynthetic rate. (**B**) Stomatal conductance to H_2_O. (**C**) Intercellular CO_2_ concentration. (**D**) Transpiration rate. N = 6. Different letters represent a significant difference between samples (*p* < 0.05). Error bars represent SE.

Compared to the control, the photosynthetic rate of the Lsa leaves subjected to 75 mM NaCl stress did not change significantly; however, it was significantly suppressed by the 150 mM NaCl treatment on Days 7 and 14 (Figure 3A). Compared to that in the control, the photosynthetic rate of the Lse leaves was significantly inhibited under both 75 mM and 150 mM NaCl, and the inhibition effect strengthened with an increasing NaCl concentration (Figure 3A). The stomatal conductance and transpiration rate of the Lsa and Lse leaves decreased significantly under 75 mM and 150 mM NaCl conditions on Day 7 (Figure 3B,D). On Day 14, the stomatal conductance of the Lsa and Lse leaves and the transpiration rate of the Lse leaves decreased significantly under the 75 mM and 150 mM NaCl treatments compared to these values in the control; in comparison, the transpiration rate of the Lse leaves subjected to 75 mM and 150 mM NaCl treatments showed no significant changes between the control and these salinity stresses (Figure 3B,D). There were no significant changes in intercellular CO_2_ in the Lsa and Lse leaves under any of the salt treatment conditions compared to those in the control on Day 7 and Day 14, apart from those in the Lsa leaves under the 150 mM NaCl stress compared to those in the control on Day 7 (Figure 3C).

**Figure 4:**
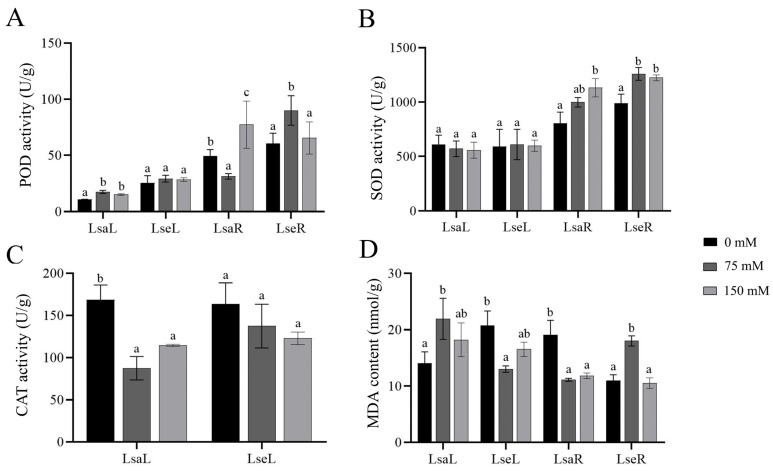
Activity of ROS-related enzymes and content of *MDA*. LsaL is *L. sativa* ‘Lollo Rosso’ leaf; LsaR is *L. sativa* ‘Lollo Rosso’ root; LseL is *L. serriola* leaf; LseR is *L. serriola* root. (**A**) *POD* activity. (**B**) *SOD* activity. (**C**) *CAT* activity. (**D**) *MDA* content. Different letters represent a significant difference between samples (*p* < 0.05). Error bars represent SE.

The antioxidant enzyme activities of superoxide dismutase (*SOD*), peroxidase (*POD*), and catalase (*CAT*) and the contents of malondialdehyde (*MDA*), proline, and soluble sugar in the lettuce leaves and roots were measured on Day 14 after NaCl treatment. The *POD* activity of the LsaL under the 75 mM and 150 mM NaCl treatments increased relative to that at 0 mM; however, there was no significant difference between the two salt treatments. The *POD* activity of the LsaR decreased under 75 mM NaCl and increased under 150 mM NaCl compared to that in the control (Figure 4A). The *POD* activity of the LseL did not clearly change under the different treatments compared to that in the control; however, that of the LseR increased under 75 mM salinity conditions compared to that in the control, and no significant change was noted under the 150 mM salinity conditions compared to that in the control (Figure 4A). The *SOD* activity in the lettuce leaves showed no significant changes among the different conditions; in comparison, the *SOD* activity significantly increased in the LsaR at 150 mM compared to in the 0 mM NaCl treatment and in the LseR under 75 mM and 150 mM NaCl conditions compared to that in the control (Figure 4B). *CAT* activity was detected in the leaves only and decreased under salt treatment in both types of lettuce leaves (Figure 4C). The maximum *MDA* content in the LsaL and LseR was achieved under 75 mM salt conditions, and the maximum concentrations of *MDA* in the LseL and LsaR were achieved under control conditions (Figure 4D).

**Figure 5:**
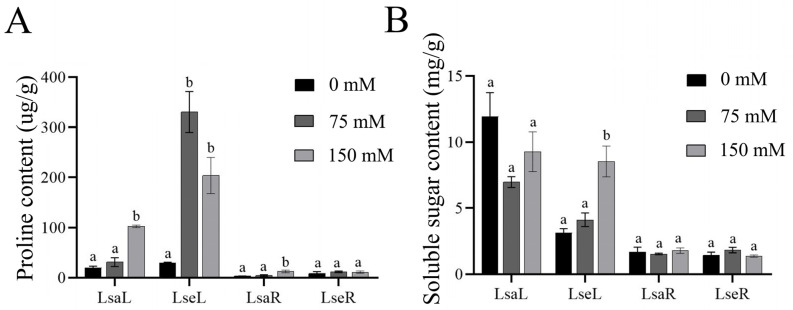
Contents of osmoregulation-related substances. LsaL is *L. sativa* ‘Lollo Rosso’ leaf; LsaR is *L. sativa* ‘Lollo Rosso’ root; LseL is *L. serriola* leaf; LseR is *L. serriola* root. (**A**) Proline content. (**B**) Soluble sugar content. Different letters represent a significant difference between samples (*p* < 0.05). Error bars represent SE.

A significant increase in the proline content under salt stress was noted in the leaves from both lettuces (Figure 5A), and the soluble sugar content of the wild lettuce leaves also increased significantly (Figure 5B).

### 2.3. The Identification of Differentially Expressed Genes in Lettuce Under Normal and Salt Treatment Conditions

A total of 24 RNA samples were successfully sequenced and generated raw data. After filtering out the low-quality data by applying stringent standards, approximately 156.86 Gb of clean data was ultimately obtained, with all Q30 scores of the clean reads > 91.81%, and the GC content of all of these samples ranged from 43.19% to 45.53%. Lastly, a reference transcriptome of these samples with a total of 47,682 transcripts was obtained. The ratio of clean reads mapped to the reference transcriptome ranged from 92.77% to 97.05% (Table S1).

**Figure 6:**
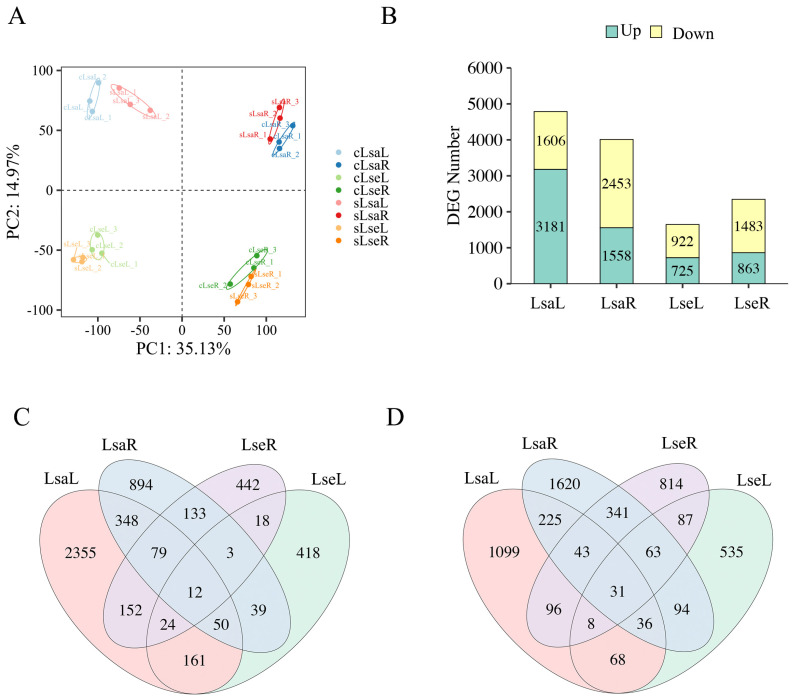
Principal component analysis (PCA) and the number of differentially expressed genes in the roots and leaves of two lettuce species under control and salt conditions. LsaL is *L. sativa* ‘Lollo Rosso’ leaf; LsaR is *L. sativa* ‘Lollo Rosso’ root; LseL is *L. serriola* leaf; LseR is *L. serriola* root. (**A**) PCA of the gene expression in the leaves and roots of two lettuce seedlings under control and salt conditions. (**B**) The number of upregulated and downregulated DEGs with a significance level of padj < 0.05, |log_2_ fold change (FC)| ≥ 1. (**C**) Venn diagram of upregulated DEGs. (**D**) Venn diagram of downregulated DEGs.

To assess the degree of variation and reproducibility among transcriptomes, a principal component analysis (PCA) was performed using FPKM data for all of the samples. The two-dimensional plot showed that the first principal component accounted for 35.13% of the total variance and clearly distinguished the different tissues. The second principal component separated the Lsa samples from the Lse samples and accounted for 14.97% of the total variance (Figure 6A).

Differential expression analyses of four paired groups, including Lsa leaves under control conditions (cLsaL), Lsa leaves under salt conditions (sLsaL), Lsa roots under control conditions (cLsaR), Lsa roots under salt conditions (sLsaR), Lse leaves under control conditions (cLseL), Lse leaves under salt conditions (sLseL), Lse roots under control conditions (cLseR), and Lse roots under salt conditions (sLseR), in the form of sLsaL vs. cLsaL, sLsaR vs. cLsaR, sLseL vs. cLseL, and sLseR vs. cLseR, were performed, with a significance level of padj < 0.05 and |log_2_ FC| ≥ 1. We identified 3181 upregulated differential expression genes (DEGs) and 1606 downregulated DEGs in the LsaLs and 1558 upregulated DEGs and 2453 downregulated DEGs in the LsaRs (Figure 6B). In Lse, a total of 725 upregulated DEGs and 922 downregulated DEGs were identified in the leaves, and 863 upregulated DEGs and 1483 downregulated DEGs were found in the roots (Figure 6B). A total of 247 upregulated DEGs and 167 downregulated DEGs were identified as being shared between the Lsa and Lse leaves, and 227 common upregulated DEGs and 478 common downregulated DEGs were found in the Lsa and Lse roots (Figure 6C,D).

### 2.4. Functional Annotation of the DEGs

To enhance our understanding of the possible functions of these DEGs, a Kyoto Encyclopedia of Genes and Genomes (KEGG) pathway enrichment analysis was also performed on the DEGs obtained. The top 20 significantly enriched KEGG pathways were illustrated, with a significance level of *p* < 0.05.

**Figure 7:**
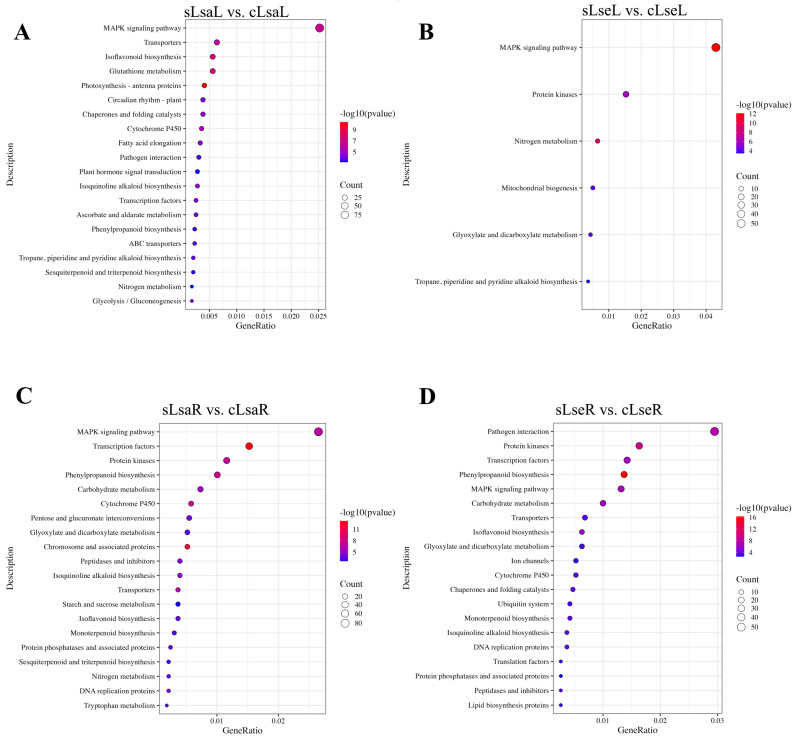
KEGG enrichment analysis of DEGs in Lsa and Lse leaves and roots. LsaL is *L. sativa* ‘Lollo Rosso’ leaf; LsaR is *L. sativa* ‘Lollo Rosso’ root; LseL is *L. serriola* leaf; LseR is *L. serriola* root. (**A**) DEGs in Lsa leaves. (**B**) DEGs in Lse leaves. (**C**) DEGs in Lsa roots. (**D**) DEGs in Lse roots. Four paired groups included Lsa leaves under control conditions (cLsaL), Lsa leaves under salt conditions (sLsaL), Lsa roots under control conditions (cLsaR), Lsa roots under salt conditions (sLsaR), Lse leaves under control conditions (cLseL), Lse leaves under salt conditions (sLseL), Lse roots under control conditions (cLseR), and Lse roots under salt conditions (sLseR), in the form of sLsaL vs. cLsaL, sLsaR vs. cLsaR, sLseL vs. cLseL, and sLseR vs. cLseR.

For the lettuce leaves, the DEGs significantly enriched in the LsaLs were related to the following: the MAPK signaling pathway, transporters, isoflavonoid biosynthesis, glutathione metabolism, photosynthesis–antenna proteins, circadian rhythm, chaperones and folding catalysts, cytochrome P450, fatty acid elongation, plant–pathogen interaction, plant hormone signal transduction, isoquinoline alkaloid biosynthesis, transcription factors, ascorbate and aldarate metabolism, phenylpropanoid biosynthesis, ABC transporters, tropane, piperidine and pyridine alkaloid biosynthesis, sesquiterpenoid and triterpenoid biosynthesis, nitrogen metabolism, glycolysis/gluconeogenesis, etc. (Figure 7A). Compared to cultivated lettuce, fewer DEGs were identified and significantly enriched for the MAPK signaling pathway, protein kinases, nitrogen metabolism, mitochondrial biogenesis, glyoxylate and dicarboxylate metabolism, tropane, and piperidine and pyridine alkaloid biosynthesis (Figure 7B).

**Figure 8:**
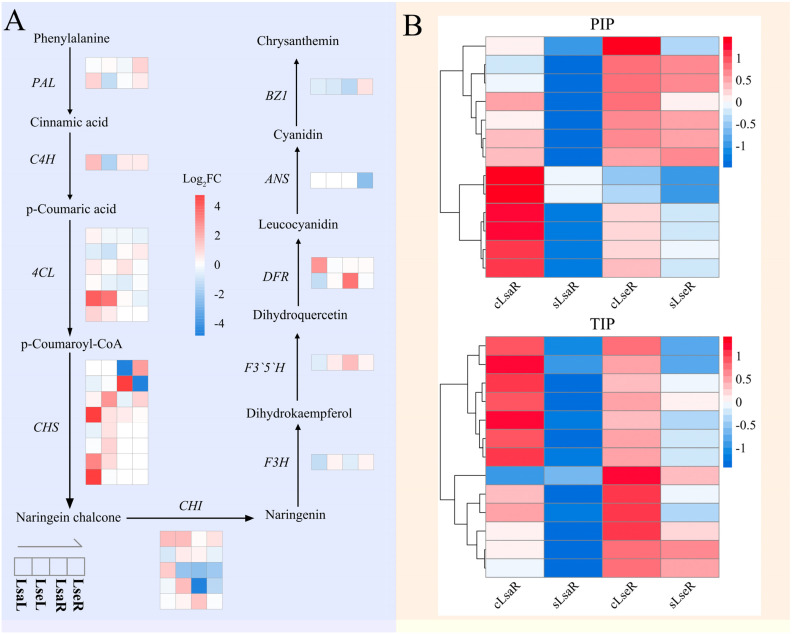
An expression heat map of DEGs related to phenylpropanoid biosynthesis, isoflavonoid biosynthesis, aquaporin *PIP* (Plasma Membrane Intrinsic Protein), and aquaporin *TIP* (Tonoplast Intrinsic Protein) in the Lsa and Lse leaves and roots in response to salt stress. LsaL is *L. sativa* ‘Lollo Rosso’ leaf; LsaR is *L. sativa* ‘Lollo Rosso’ root; LseL is *L. serriola* leaf; LseR is *L. serriola* root. (**A**) DEGs related to phenylpropanoid biosynthesis and isoflavonoid biosynthesis. (**B**) Expression heat maps of *PIP-* and *TIP*-related DEGs. The color scale shows the row-normalized Z-scores computed from the FPKM values (red: high expression; blue: low expression).

In the lettuce roots, the enriched DEGs in the LsaRs were mainly related to the MAPK signaling pathway, transcription factors, protein kinases, phenylpropanoid biosynthesis, carbohydrate metabolism, cytochrome P450, pentose and glucuronate interconversions, glyoxylate and dicarboxylate metabolism, chromosome and associated proteins, peptidases and inhibitors, isoquinoline alkaloid biosynthesis, transporters, starch and sucrose metabolism, isoflavonoid biosynthesis, monoterpenoid biosynthesis, protein phosphatases and associated proteins, sesquiterpenoid and triterpenoid biosynthesis, nitrogen metabolism, DNA replication proteins, and tryptophan metabolism (Figure 7C). The significantly enriched DEGs in the LsaRs were related to plant–pathogen interactions, protein kinases, transcription factors, phenylpropanoid biosynthesis, the MAPK signaling pathway, carbohydrate metabolism, transporters, isoflavonoid biosynthesis, glyoxylate and dicarboxylate metabolism, ion channels, cytochrome P450, chaperones and folding catalysts, the ubiquitin system, monoterpenoid biosynthesis, isoquinoline alkaloid biosynthesis, DNA replication proteins, translation factors, protein phosphatases and associated proteins, peptidases and inhibitors, and lipid biosynthesis proteins (Figure 7D). The DEGs for the KEGG pathways related to phenylpropanoid biosynthesis, isoflavonoid biosynthesis, aquaporin *PIP* (Plasma Membrane Intrinsic Protein), and aquaporin *TIP* (Tonoplast Intrinsic Protein) in the two lettuce species are shown in a heat map (Figure 8).

### 2.5. The Key DEGs Involved in Salt Stress

In all of the samples, certain key genes related to ROS scavenging were significantly upregulated in their expression. *POD* was significantly upregulated in the leaves and roots of Lsa and the roots of Lse under salt treatment (Figure S1). *SOD* was significantly upregulated in both the leaves and roots of Lsa and Lse under salt treatment. The *GST* gene expression was significantly elevated in the Lsa leaves and the Lse roots under salinity stress. P5CS and OAT are key enzymes in the synthesis of proline from glutamate or ornithine [20], respectively. OAT was significantly upregulated in the Lsa leaves only, and the *P5CS* expression was significantly elevated in both the Lsa and Lse leaves and roots (Figure S1). ABA-mediated *SnRK2*, JA-mediated *JAR4*, and SA-mediated *NPR1* were significantly upregulated under salt treatment in the Lsa leaves, with one *SnRK2.7* upregulated in the Lsa roots (Figure S1).

### 2.6. qRT-PCR Validation of the DEGs

**Figure 9:**
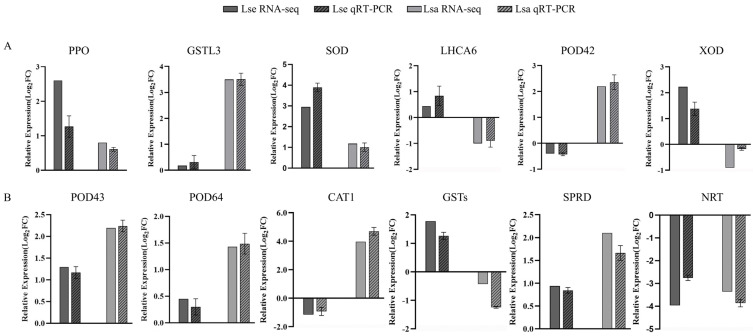
Validation of RNA-Seq results via qRT-PCR. Lsa is *L. sativa* ‘Lollo Rosso’; Lse is *L. serriola*. (**A**) Gene expression in lettuce leaves and (**B**) gene expression in lettuce roots.

Twelve DEGs were selected to verify the reliability of the transcriptomic data. Up- and downregulated expression levels of *PPO*, *GSTL3*, *SOD*, *LHCA6*, *POD42*, and *XOD* were identified in the Lsa and Lse leaves, whereas those of *POD43*, *POD63*, *CAT1*, *GSTs*, *SPRD*, and *NRT* were identified in the Lsa and Lse roots (Figure 9). In general, the qPCR and RNA-seq data were consistent in terms of the expression levels.

## 3. Discussion

In this study, we evaluated the phenotypic and physiological responses to salinity stress in cultivated and wild lettuce seedlings and explored the different gene expression levels in these two species. In general, the growth and development, net photosynthetic rate, and stomatal conductance of Lsa and Lse were shown to be inhibited by salt stress, consistent with the results of previous studies on the physiological changes in lettuce under salt stress [21,22]. The plant’s photosystem comprises photosystems I and II and contains different light capture complexes (also referred to as antenna proteins) [23]. The gene expression of Lsa antenna proteins was found to be repressed in the leaves, compromising the ability of Lsa to capture light and in turn leading to decreased photosynthesis. Salt stress leads to the production of ROS in plants, which impacts the composition and permeability of the plant plasma membrane, resulting in the disruption of normal cell membrane functions. *SOD*, *POD*, *CAT*, *GPX*, and *GST* are common ROS-scavenging-related enzymes found in plants [24]. *MDA* is one of the final products of polyunsaturated fatty acid peroxidation in the cells and is a widely used and reliable marker for determining the degree of injury to a stressed plant. In one study, increased activities of antioxidant enzymes such as *SOD*, *POD*, and *CAT* in cotton were found to reduce the damage caused by light salt stress [25]. In another study, the *POD*, *SOD*, *CAT*, *GPX*, and *GST* gene expression of salt-tolerant varieties of barley was significantly upregulated under salt conditions compared with that in sensitive varieties [26]. In the present study, the *POD*, *SOD*, *CAT*, *GPX*, and *GST* gene expression levels were upregulated in Lsa and Lse under salt stress conditions to counteract the oxidative stress caused by external salt stress. The enriched DEGs in red lettuce leaves were also related to glutathione metabolism, suggesting cellular responses to adapt to salt stress. Proline and soluble sugars are common osmoregulatory substances in plants that are utilized to alleviate the osmotic stress produced by salt ions [27]. The soluble sugar content can balance the internal and external osmotic pressure in cells to protect rice cultivars under salt stress conditions, with the plants able to absorb water and transport nutrients from underground [28]. Our results demonstrate that both cultivated (Lsa) and wild lettuce (Lse) can increase its proline content by upregulating its gene expression of proline synthase genes (*P5CS* and *OAT*) to reduce osmotic pressure and relieve salt stress. Wild lettuce (Lse) can increase the soluble sugar content in its leaves to adjust to external salt stress.

In addition to phenotypic and physiological responses to salinity, there are five pathways commonly shared by these two lettuce germplasms that may aid them in adapting to or alleviating salt stress, namely the mitogen-activated protein kinase (MAPK) signaling pathway, transporters, cytochrome P450, phenylpropanoid biosynthesis, and isoflavonoid biosynthesis. MAPK cascades are essential for normal plant growth and development, plant immunity, and the response to environmental stresses, such as high salinity, drought, and extreme temperatures, and can convert stress signals into cellular responses in plants to adjust their metabolism, growth, and development to maintain their survival and reproduction [29]. The MAPK pathway functions in concert with cytosolic and nuclear events to maintain ionic homeostasis and signal transduction under the salt stress response in *Arabidopsis* [30]. *OsMPK4* has been reported to promote the phosphorylation and degradation of Ideal Plant Architecture 1 (IPA1) under salt stress conditions to confer salt tolerance in rice [31].

Proton oligopeptide cotransporters were enriched among the transporters in the LsaL, LsaR, and LseR. Aquaporin *PIP*, aquaporin *TIP*, and vacuolar iron transporter family protein were enriched in the transporter pathway in the LsaR. Aquaporins (AQPs) are responsible for transmembrane water transport in plants [32]. Plasma-membrane-localized aquaporin *AtPIP2;1* was found to confer resistance to disease and salt stresses, and hydrogen peroxide (H_2_O_2_) treatment in Arabidopsis downregulated the expression of the *AtPIP2* subfamily in its roots but not in its leaves [33,34]. Salinity treatment increased the protein levels of the aquaporins *HaPIPs* and *HaTIPs* in sunflower (*Helianthus annuus* L.) roots; in comparison, the exogenous application of NO downregulated their abundance, suggesting a correlation between AQPs and ion homeostasis in response to salt stress and NO [35]. The downregulated expression of the aquaporins *PIP* and *TIP* in the LsaR suggest a response to salt stress in order to decrease H_2_O and H_2_O_2_ transport. From the above results, it is evident that cultivated and wild lettuce roots show different DEG responses in terms of their levels of transporters.

Lettuce leaves contain numerous phytochemicals that relate to the leaves’ color, flavor, nutritional components, and bioactivity, such as glycosylated flavonoids, phenolic acids, carotenoids, vitamin B groups, ascorbic acid, tocopherols, and sesquiterpene lactones [36]. The most common phenolic acids in lettuce are caffeic acid and chlorogenic acid derivatives, and the most common flavonoids in lettuce are quercetin and kaempferol derivatives, anthocyanins, and flavone luteolin [37]. Red lettuce leaves are rich in phenolic acids, anthocyanins, and carotenoids [37]. The contents of these phytochemical compounds and the antioxidant capacity of red lettuce cultivars are reported to be affected by the growing season, and phenolic compounds, in particular anthocyanins, are positively correlated with antioxidant activity [38]. The results of the KEGG analysis revealed that the enriched DEGs in the cultivated red lettuce and wild species were related to isoflavonoid biosynthesis under salt stress. The upregulated DEGs related to phenylpropanoid/isoflavonoid biosynthesis in the two lettuce species indicated that this secondary metabolism responded to salinity stress in the lettuce. The number of DEGs in red lettuce was greater than that in wild lettuce, with the former showing stronger physiological responses than those in the wild species.

Plant isoflavonoids are phenolic metabolites that are derived from the phenylpropanoid pathway. At present, an increasing number of studies have highlighted the vital role of phenylpropanoid metabolism in plant development and plant–environment interplay. Phenylpropanoid metabolism generates more than 8000 metabolites, including lignin, phenylpropanoid esters, flavonoids, lignans, hydroxycinnamic acid amides, and sporopollenin [39]. Phenolic metabolites in higher plants are primarily biosynthesized by the shikimic acid pathway, which is the precursor for the synthesis of aromatic acids such as phenylalanine, tyrosine, and tryptophan [40]. Simple phenolic acids impact the plasma membrane, cellular respiration and photosynthesis, lignin metabolism, water relations, hormonal balance, oxidative stress, and related enzymes in plants [41]. Flavonoids have been reported to protect plants against biotic and abiotic stresses, such as pests and diseases, ultraviolet light, cold, and salinity [42,43,44], in addition to scavenging the ROS and free radicals produced during salt stress [43,45]. In a recent study, a multi-omics analysis of the salt stress response in rosa plants revealed that the phenylpropane pathway, patricianly flavonoid and flavonol metabolism, is strongly enhanced in roses under salt stress, and *chalcone synthase 1* (*CHS1*) was identified as the key regulatory gene [43]. The halophyte *Limonium bicolor* was reported to suffer from severe osmotic and oxidative stress under high-salt-stress conditions and alleviate salt stress by increasing the abundance of organic soluble substances and flavonoids [46]. The results of a study on the impact of drought stress on the antioxidant (vitamin C and anthocyanin) content in cultivated lettuces and wild relatives (*Lactuca dregeana* DC. and *Lactuca homblei* De Wild) demonstrated an increase in the total anthocyanin content in the leaves of red commercial varieties and wild relatives, indicating that anthocyanins may play a role in the mechanisms deployed by these plants to tolerate drought stress [47].

In addition to phenylpropanoid and isoflavonoid biosynthesis, the other secondary metabolism pathways in the cultivated and wild lettuces also responded to salinity stress. The superfamily of Cytochrome P450 (*CYP*) contains a significant number of enzymes that play a key role in plants’ evolution and the biosynthetic pathways of metabolites such as flavonoids, terpenoids, steroids, cyanogenic glucosides and volatile nitriles, furanocoumarins, and momilactones [48,49]. Isoquinoline alkaloid biosynthesis, monoterpenoid biosynthesis, and sesquiterpenoid and triterpenoid biosynthesis are involved in plant defense, growth, and development [50]. These pathways are also enriched in cultivated and wild lettuce germplasms. Exogenous monoterpenes have been reported to alleviate H_2_O_2_-induced lipid damage during water deficits in tomato [51]. From the above findings, it can be concluded that phenylpropanoid and isoflavonoid biosynthesis and the *CYP* gene family in these two lettuce species are important secondary metabolism pathways that respond to salinity stress in lettuce.

## 4. Materials and Methods

### 4.1. The Plant Materials and Salt Stress Treatments

Cultivated lettuce, *Lactuca sativa* L. ‘Lollo Rosso’ (Lsa), seeds were purchased from a local market, and wild lettuce, *Lactuca serriola* L. (Lse), seeds were collected on the campus of Zhengzhou University, China. The lettuce seeds were germinated in a substrate at 22 °C with 50% humidity and a photoperiod of a 16/8 h light–dark cycle. Lettuce seedlings with five true leaves and similar leaf areas were transferred into cases with 10 L of Hoagland’s nutrient solution for 5 days to allow them to adapt to the hydroponic system and then treated with different NaCl concentrations (0, 75, and 150 mM NaCl) for two weeks. Fresh leaf and root materials were collected from the lettuce treated with 0 and 150 mM NaCl after two weeks, immediately frozen in liquid nitrogen, and stored in an −80 °C freezer for RNA isolation.

### 4.2. Measurements of Their Phenotypic and Photosynthetic Traits, Antioxidant Enzymatic Activity, and Proline and Soluble Sugar Contents

The leaves and roots of the lettuce were photographed (Nikon D750, Thailand) on Days 0, 7, and 14 after the salinity treatment. LA and MRL were measured using ImageJ v1.53v [52]. The net photosynthetic rate, stomatal conductance, intercellular CO_2_ concentration, and transpiration rate of both types of lettuce leaves were measured using an LI-6400 portable photosynthesis measurement system (LI-COR Biosciences, Lincoln, NE, USA) from 9 a.m. to 2 p.m. on Day 0, Day 7, and Day 14 after treatment. These phenotypic and photosynthesis-related parameters were measured in 10 biological replicates. The activities of superoxide dismutase (*SOD*), peroxidase (*POD*), and catalase (*CAT*) and the contents of malondialdehyde (*MDA*), proline, and soluble sugars in the leaves and roots of the cultivated and wild lettuce seedlings after Day 14 of the treatment were determined following the manufacturer’s instructions (M0102A, M0105A, M0103A, M0301A, M0106A, M0108A, M1503A, Suzhou Michy Biomedical Technology, Suzhou, Jiangsu, China), including three biological replicates and three technical repeats for each sample.

### 4.3. RNA Extraction, Library Preparation, and RNA Sequencing

The total RNA samples from the root and leaf materials of the lettuce (three biological replicates for each type) under the control and salt stress (150 mM NaCl) conditions were extracted using the FastPure Plant Total RNA Isolation Kit (RC401-01, Vazyme Biotech, Nanjing, China). The concentrations of the RNA samples were determined using a NanoDrop 2000 spectrophotometer, model A260/A280 (Thermo Fisher Scientific, Wilmington, DE, USA), and the quality of the RNA was assessed using 1% agarose gel electrophoresis. The integrity of the RNA samples was assessed based on the RNA Nano 6000 Assay Kit for the Agilent BioAnalyzer 2100 system (Agilent Technologies, Santa Clara, CA, USA). mRNA was enriched from the total RNA using oligonucleotide (dT)-linked magnetic beads and subsequently randomly interrupted with divalent cations in NEB Fragmentation Buffer. The first-strand cDNA was synthesized using random hexamer primers and M-MuLV reverse transcriptase. Second-strand cDNA synthesis was performed using DNA polymerase I and RNase H, and double-strand cDNA was end-repaired. Adenosine was added to the ends and ligated to the adapters. AMPure XP beads were applied to screen fragments in the 250–300 bp size range. The obtained cDNA was subjected to PCR amplification and then purified using AMPure XP beads to obtain the final library. Paired-end 2× 150 bp reads were sequenced using the NovaSeq 4000 platform (Novogene Co., Ltd., Tianjin, China).

### 4.4. Quality Control and Transcriptome Assembly

The quality of the raw RNA-seq data was assessed using the FastQC (version 0.11.9) (http://www.bioinformatics.babraham.ac.uk/projects/fastqc/, accessed on 1 September 2024) program. Low-quality bases and the adapters at the 3′ end were removed using Trim Galor (version 0.6.5) (https://www.bioinformatics.babraham.ac.uk/projects/trim_galore/, accessed on 1 September 2024) to obtain clean reads. All of the clean reads were aligned with the reference genome for lettuce (https://ftp.ncbi.nlm.nih.gov/genomes/all/GCF/002/870/075/GCF_002870075.4_Lsat_Salinas_v11/, accessed on 1 October 2024) using HISAT2 2.2.1 [53] with the default parameters. Mapped reads were assembled and quantified using StringTie 2.2.1 [54].

### 4.5. The Identification of Differential Expression Genes and the Functional Enrichment Analysis

The RNA-seq data were divided into eight groups: Lsa leaves under control conditions (cLsaL), Lsa leaves under salt conditions (sLsaL), Lsa roots under control conditions (cLsaR), Lsa roots under salt conditions (sLsaR), Lse leaves under control conditions (cLseL), Lse leaves under salt conditions (sLseL), Lse roots under control conditions (cLseR), and Lse roots under salt conditions (sLseR). Each treatment included three biological replications. Differential expression analyses of four paired groups (sLsaL vs. cLsaL, sLsaR vs. cLsaR, sLseL vs. cLseL, and sLseR vs. cLseR) were performed using the DESeq2 package in R [55]. Genes with |log_2_ FC| > 1 and a *p*-value < 0.05 were considered to be significantly differentially expressed.

The KEGG pathway enrichment analysis of the differentially expressed genes (DEGs) was performed using the clusterProfiler package [56] in R based on the hypergeometric test.

### 4.6. Quantitative Real-Time PCR Validation

To validate the RNA-seq data, twelve DEGs were randomly selected for qRT-PCR, and their primers were designed using Primer-BLAST (https://www.ncbi.nlm.nih.gov/tools/primer-blast/, accessed on 1 December 2024) and synthesized by Sangon Biotech (Shanghai) Co., Ltd. (Shanghai, China) (Table S2). Roughly 1.0 μg of total RNA was used to obtain cDNA using PrimeScript™ RT Master Mix (Vazyme, Nanjing, China), following the manufacturer’s instructions. QRT-PCR was performed using an UltraSYBR Mixture (Low ROX) Kit (CWBIO, Beijing, China) with 1 µL of cDNA template and a total 25 µL reaction volume with three biological replicates and two technical replicates, following the manufacturer’s instructions. Tubulin (*TUB*) and actin (*ACT*) were used as the internal control genes to normalize the expression levels (Table S2). Relative expression levels were calculated using the 2−ΔΔCT method [57].

### 4.7. Statistical Analysis

All of the data are shown as the means ± standard error of the mean (SEMs) of at least three independent replicates. A one-way ANOVA followed by Tukey’s honestly significant difference test was used to analyze the data. The level of statistical significance was a *p*-value < 0.05.

## 5. Conclusions

In this study, the phenotypic, physiological, and transcriptomic changes in wild and cultivated lettuce were measured and analyzed under different salinity treatments. The growth, development, and photosynthesis of Lsa and Lse were inhibited under salt stress. The proline content was increased in the Lsa and Lse leaves, with upregulated expression of proline synthase genes (*P5CS* and *OAT*). The soluble sugar content in the Lse leaves increased significantly to adjust to external salt stress. The MAPK signaling pathway, transporters, cytochrome P450, phenylpropanoid biosynthesis, and isoflavonoid biosynthesis were identified as key pathways in the lettuce seedlings’ adaptation to salt stress. The red lettuce cultivar showed a comparable tolerance capacity to that of the wild lettuce species under salt stress through upregulated DEGs in the aquaporin and biosynthesis pathways for antioxidant compounds.

## Data Availability

The datasets generated and/or analyzed in this study can be found in the NCBI database under the BioProjects IDs PRJNA1037362 and PRJNA1045380.

## Supplementary Materials

The following supporting information can be downloaded at https://www.mdpi.com/article/10.3390/ijms26073425/s1.

## Abbreviations

The following abbreviations are used in this manuscript:

Lsa	*L. sativa* ‘Lollo Rosso’
Lse	*L. serriola*
LsaL	*L. sativa* ‘Lollo Rosso’ leaf
LsaR	*L. sativa* ‘Lollo Rosso’ root
LseL	*L. serriola* leaf
LseR	*L. serriola* root
LA	Leaf area
LDW	Leaf dry weight
MRL	Main root length
RDW	Root dry weight
