# Supplementary material for: Phenotypic, Physiological, and Transcriptomic Analyses Reveal Different Responses to Salt Stress in Cultivated Red Lettuce and Wild Lettuce Seedlings
Source: Int J Mol Sci. 2025 Apr 6;26(7):3425. doi: 10.3390/ijms26073425 (PMC11989937; doi:10.3390/ijms26073425)
Supplement: Supplementary file 1 [file ijms-26-03425-s001.zip › ijms-3526053-supplementary.pdf]

Table S1 Summary of transcriptome assemblies

Sample	Raw_Reads	Clean_Reads	Clean_Bases	Error_Rate	Q30	GC_pct	Total Map
cLsaL_1	48870436	47697172	7.15G	0.03	93.73	44.64	96.43%
cLsaL_2	44868542	43128590	6.47G	0.03	93.36	44.3	97.02%
cLsaL_3	42708946	41210808	6.18G	0.03	93.13	44.5	96.88%
sLsaL_1	43205740	41534076	6.23G	0.03	92.98	44.55	96.84%
sLsaL_2	44284696	42494052	6.37G	0.03	93.32	44.38	97.05%
sLsaL_3	46750072	44415090	6.66G	0.03	92.39	44.35	96.02%
cLsaR_1	44965336	42294924	6.34G	0.03	93.23	43.38	96.41%
cLsaR_2	44446892	42591772	6.39G	0.03	92.97	43.2	96.46%
cLsaR_3	42354972	39775186	5.97G	0.03	93.65	43.25	96.78%
sLsaR_1	45412564	42359982	6.35G	0.03	93.46	43.82	94.97%
sLsaR_2	43538572	40597070	6.09G	0.03	93.32	43.55	95.36%
sLsaR_3	44690828	42045202	6.31G	0.03	93.07	43.19	94.01%
cLseL_1	45395186	44329146	6.65G	0.03	92.57	44.77	94.60%
cLseL_2	40261058	39673456	5.95G	0.03	92.22	44.65	94.58%
cLseL_3	44847996	44053680	6.61G	0.03	92.24	45.27	94.57%
sLseL_1	45685872	44587026	6.69G	0.03	92.25	45.03	94.88%
sLseL_2	46398618	45829284	6.87G	0.03	92.29	45.27	95.23%
sLseL_3	50174800	49108934	7.37G	0.03	92.41	45.53	94.96%
cLseR_1	42221806	41362660	6.2G	0.03	91.81	44.27	93.60%
cLseR_2	42094786	41368930	6.21G	0.03	92.09	44.2	93.80%
cLseR_3	59041384	57898944	8.68G	0.03	91.9	44.01	91.34%
sLseR_1	44864494	44034592	6.61G	0.03	92.08	45.16	91.55%
sLseR_2	44400902	43596816	6.54G	0.03	92.14	45.45	92.80%
sLseR_3	40702014	39824490	5.97G	0.03	91.83	43.81	92.29%

Table S2 Primer sequences

Gene symbol	Primer sequence (5′-3′) (Forward/Reverse)
ACT	AGGGCAGTGTTTCCTAGTATTGTTG/CTCTTTTGGATTGTGCCTCATCT
TUB	TAGGCGTGTGAGTGAGCAGT/AACCCTCGTACTCTGCCTCTT
GSTL3	GGAACAACAAGGTTATACACG/AAATCTAAGCTCTCTCCGATG
POD42	GATTCATGCCCACAAGCTG/ATTTCTAAGTCCGAAGCTCCG
LHCA6	ACTCCTCCTGAATGGCTTGAT/TCTCTTGACCCAGCATCGAAC
CAT1	CGAAACTCATCCTCCCTCCC/ACCCTTTGCACTAGCACCTC
SPRD	GATTGGATGCTCCGGTGGAA/GCGAAGAGGAAGCAGATGGA
PPO	ATCCTCTCTTTTACGTCCACC/CGTAGTCATATCCCATCCGA
SOD	TCCTTTTCCCCATCTCAAACC/AGAGTAACAACGCCTTCGAC
XOD	TCAACACTATACCGCAACACC/CCCATCAAACATGCCCAAAC
GSTs	CTGCACAATGGAAATCCGA/AATTTCGCCCAGAATCTAGC
NRT	TGTCCACCTCTCCGCACTTA/GTGATGTCAAAGGGGCAGGT
POD43	ATGCAGCCAATTCTAACCCTC/AACACTTTCCAACTTCGCTTT
POD63	CGTCGTTCAATCCAAGATACGC/CTCCAAGTCCTTTCGGCAAG

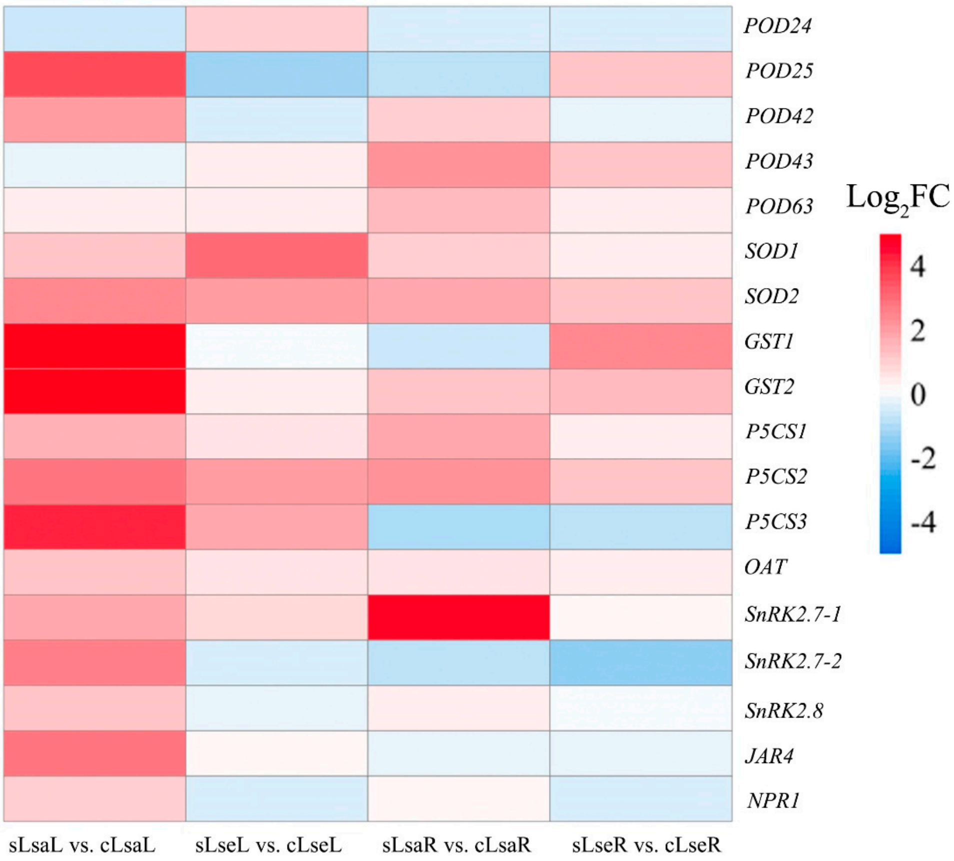

Figure S1. Functional annotation of some differentially expressed genes related to salt stress in two lettuce species. Four paired groups included Lsa leaf under control conditions (cLsaL), Lsa leaf under salt conditions (sLsaL), Lsa root under control conditions (cLsaR), Lsa root under salt conditions (sLsaR), Lse leaf under control conditions (cLseL), Lse leaf under salt conditions (sLseL), Lse root under control conditions (cLseR), and Lse root under salt conditions (sLseR). in the form of (sLsaL vs. cLsaL, sLsaR vs. cLsaR, sLseL vs. cLseL, and sLseR vs. cLseR).
